# Becoming Self Employed: Israeli Family Physicians’ Push and Pull Factors

**DOI:** 10.3390/healthcare12171749

**Published:** 2024-09-02

**Authors:** Irit Chudner, Avi Shnider, Omer Gluzman, Hadas Keidar, Motti Haimi

**Affiliations:** 1 School of Behavioral Sciences, College of Management Academic Studies, Rishon LeZion 7570724, Israel; 2 Maccabi Healthcare Services, Tel Aviv 6801296, Israel; 3 Health Systems Management Department, Yezreel Valley College, Yezreel Valley 1930600, Israel; 4 Rappaport Faculty of Medicine, Technion-Israel Institute of Technology, Haifa 3109601, Israel; 5 Meuhedet Health Services, Tel Aviv 6203854, Israel

**Keywords:** primary care physicians, pull factors, push factors, managed care organization, self-employed

## Abstract

Background: As primary care is an important infrastructure for the entire health system, the employment structure choices of family physicians—whether to work in a managed care organization or be self-employed, can impact all effectiveness parameters of healthcare: quality, access, health equity, patients’ experiences, and cost-effectiveness. The aim of this study is to assess the push and pull factors influencing family physicians’ employment choices. Methods: This study employed a qualitative approach to explore the experiences of family physicians (FPs) who choose to work a self-employment practice. We conducted semi-structured interviews with twenty-seven self-employed FPs in Israel, selected through purposive and snowball sampling. The interviews were conducted via Zoom, recorded, and transcribed verbatim. Data analysis followed thematic analysis framework. The analysis yielded 10 themes, which were organized into two categories: pull and push factors. Results: Pull factors, i.e., factors that attract family physicians to become self-employed, included professional self-fulfillment, higher income, professional and business autonomy, working with secretaries according to one’s personal choice, designing the space of the clinic, and flexibility in working hours. Push factors, i.e., factors that demotivated family physicians to work under managed care and pushed them to choose self-employment included low control over the work environment, workload, decreased professional and organizational autonomy, managerial pressures on quality measures, engagement in marketing activities, and tensions with non-medical staff in the Health Maintenance Organization. Conclusions: There are obvious implications of this work for Health Maintenance Organizations’ policy makers. Balancing managerial pressure and tensions between family physicians and non-medical administration and ensuring suitable working conditions increased physicians’ control over the work environment, and professional autonomy may decrease push factors and retain family physicians as Health Maintenance Organization-employed. Understanding pull factors may help to develop a strategy for maximizing cooperation with self-employed family physicians and reinforce physicians’ linkage to the healthcare system’s treatment objectives.

## 1. Introduction

Family physicians (FPs) play a key role in guaranteeing access to health care, managing chronic conditions, and keeping patients out of hospital [1,2,3,4]. Labor markets in primary healthcare systems vary around the world. They are built on different combinations of FP practices. These practices include both employed and self-employed family physicians. As primary care is an important infrastructure for the entire health system, FPs’ employment structures can impact the health quality and the effectiveness of the entire health system [2,3]. The divergence between HMO-employed and the self-employment FP models bears implications for patient care, FP efficacy and burnout, and the professional perception of the FP’s role [2,5,6]. The FP’s role is traditionally considered one of trust, wherein patients rely on their FP to adhere to their needs and interests. Within HMOs, FPs may encounter conflicts of interest due to their obligations to both the patient and the organization. In contrast, self-employed FPs might be less encumbered by such organizational constraints. Additionally, compensation structures vary between these employment models. HMO employment typically utilizes capitation, wherein FPs are remunerated based on the number of patients they oversee [1,7]. In contrast, self-employment often uses a fee-for-service system. This latter approach may promote greater efficacy in patient care, but also limits the HMO’s ability to manage self-employed FPs. Furthermore, self-employment entails management responsibilities for one’s own clinic, affording greater autonomy and decision-making authority, whereas in managed care, administrative duties are usually centralized within the HMO. 

This movement from being employed by an HMO to becoming self-employed can challenge healthcare systems, or at the very least, impose the need for an appropriate re-design. These trends can be combined with global trends like the shortage of family physicians [7,8], aging [1,6], decreased work satisfaction [9,10], fewer medical students choosing family medicine as a specialty, and an increasing population and demand for healthcare services [8,9]. 

Considering the above-mentioned trends, and the fact that the FP labor market in the short and medium terms is fixed and inflexible [11], health systems may be significantly affected by these employment framework changes. 

This study aims to assess the push and pull factors influencing family physicians’ decisions to work in a self-employed practice in Israel. This research is significant as it addresses a critical gap in understanding physician employment preferences, which directly impacts primary care delivery. By identifying factors that motivate or demotivate family physicians in different employment models, this study provides valuable insights for health policy makers and healthcare organizations. These findings can help design more efficient and long-lasting primary care models by informing tactics to raise physician satisfaction, retention, and, eventually, patient care quality.

Given an in-depth understanding of FPs’ considerations of whether to choose a self-employment practice or to be employed by an HMO, policy makers can mitigate negative effects and increase positive effects, thus leading health systems to their optimal balance and effectiveness.

### 1.1. Self-Employment Trends in Primary Care Delivery in Israel

Self-employment, for the purposes of this study, can be defined as having an ownership stake in one’s medical practice. This implies that a share of the profits constitutes a meaningful portion of the self-employed FP’s earnings [1]—both in solo and group model practices. In Israel, out of about 5000 FPs, approximately 35% are self-employed, while the remaining 65% are HMO-employed [12,13]. HMO-employed physicians work directly for Health Maintenance Organizations like Clalit or Maccabi, typically receiving a fixed salary with set working hours and benefits. In contrast, self-employed physicians operate their own clinics, often contracting with multiple HMOs. To illustrate the difference in compensation, consider a clinic serving 2000 patients per month. An HMO-employed physician might receive a fixed monthly salary, regardless of the exact number of patients seen. A self-employed physician, however, could earn based on “capitation” (number of patients) and fee-for-service, resulting in a monthly income that could vary significantly based on actual patient volume and services provided. These differences ultimately influence the overall structure and effectiveness of primary care in Israel, affecting how physicians manage their clinics and interact with patients.

Israel provides all citizens with universal coverage as proscribed by its National Health Insurance Law. Patients are covered by a mandated benefit package encompassing a wide range of healthcare services, including hospital care, primary and specialty care, mental health, maternity care, and prescription drugs [13,14]. A self-employed FP can work under personal contract with one of the four public HMOs: Clalit, Maccabi, Meuhedet, or Leumit Health Care Services, and provide care for those organizations’ insured patients. Self-employed FPs’ reward method is based on the number of patients enrolled, new visits per month or quarter, and financial compensation in the method of payment for service on procedures [15,16]. Self-employed FPs choose their preferred type of practice—solo or group—and manage their clinics independently for all intents and purposes.

### 1.2. Push and Pull Factors Associated with FPs’ Choice to Become Self-Employed

To better understand drivers of FPs’ choice to become self-employed, the push and pull theory, initially introduced by Lee in 1966 [17]—may provide a useful lens through which to examine the phenomena. Another study developed by Kirkwood (2009) [18] employing the push and pull theory of entrepreneurship explains entrepreneurial motivations, in which their theory posits that individuals are either “pushed” into self-employment by negative factors in their current situation or “pulled” by the attractive aspects of self-employment. In the context of FPs, push factors might include dissatisfaction with bureaucratic constraints in employed positions or lack of autonomy in clinical decision-making. *Pull factors* motivate and attract workers to become self-employed, while *push factors* are those that demotivate workers to choose to be organization-employed. In general, higher income, better infrastructure, and increased autonomy are known as factors that pull family physicians into self-employment [19,20]. Known push factors include low income, work overload, and staffing shortages [19,21]. In Israel, due to a historical market structure that included mostly salaried FPs, the population of self-employed FPs remains understudied; thus, there is still not enough information about FPs’ self-employment-related push and pull factors.

In addition, the COVID-19 pandemic has significantly influenced FP employment preferences and practice patterns. According to the 2021 American Academy of Family Physicians (AAFP) Member Profile Survey, 41% of FPs reported providing telehealth services, a substantial increase from pre-pandemic levels. The same survey found that 29% of FPs experienced a decrease in work hours due to the pandemic [22]. A separate study published in the Annals of Family Medicine (2021) reported that 16% of primary care clinicians considering leaving their jobs within the next year, citing stress and burnout as major factors [23]. Furthermore, a 2022 report by the Physicians Foundation revealed that 18% of physicians changed their practice settings as a result of COVID-19 experiences, with many moving towards employed positions or different practice models [24]. These findings underscore the pandemic’s profound impact on FP work preferences, highlighting a trend towards more flexible work arrangements and a reconsideration of traditional practice models. “In addition, pandemic-related workloads in primary care and new telemedicine possibilities may affect FPs’ push and pull factors regarding the structure of their employment, but at the current time, the full extent of these influences is still unknown. To better understand why FPs decide to become self-employed, this study aimed to examine the “push and pull” factors of FPs in Israel.

## 2. Methods

### 2.1. Study Design and Sample

The decision to become self-employed was examined from a personal perspective using qualitative research methods [25]. We conducted twenty-seven semi-structured individual interviews with FPs. The participants had met two criteria: they had decided to become self-employed within the last seven years, and they were currently working in either a solo or group practice. The criterion of self-employment within the last 7 years was determined to focus on recent transitions, reflecting current trends in the medical job market while ensuring accurate recall of decision-making factors. This timeframe balanced experience with relevance to recent healthcare system changes, provided a sufficient sample size, and avoided introducing historical factors less pertinent to the present situation, though we acknowledge this as a potential limitation of this study.

Snowball sampling was used to recruit FPs, and later, purposeful sampling was added to ensure that responders’ characteristics are varied in terms of age, region, and the HMOs they work with. We chose these methods for their complementary strengths in qualitative research. Snowball sampling was initiated by contacting FPs known to the research team who met our inclusion criteria. These initial participants then referred us to other potential participants. This method was particularly useful in accessing the sometimes hard-to-reach population of self-employed FPs. Purposeful sampling was used concurrently to ensure diversity in our sample. We specifically varied the following characteristics. We recruited FPs across different age groups to capture varying career stages and generational perspectives. Participants were selected from different geographical areas within Israel, including urban and rural settings, to account for regional variations in healthcare delivery and market conditions. We ensured representation from FPs affiliated with different Health Maintenance Organizations in Israel (e.g., Clalit, Maccabi, Leumit, Meuhedet) to capture diverse organizational contexts. As this study progressed, we used purposeful sampling to fill gaps in our participant profile, ensuring a balanced representation across these characteristics. For example, when we noticed an overrepresentation of urban FPs, we specifically sought out FPs from rural areas. The combination of these methods allowed us to leverage existing professional networks (through snowball sampling) while maintaining a diverse and theoretically relevant sample (through purposeful sampling). This approach helped us gather rich, varied data that captured the complexity of FPs’ experiences with self-employment across different contexts.

The sample size was set using the “data saturation” principle. Data were collected until the additional data raised in interviews no longer contributed to the understanding of the FPs’ push and pull factors. To determine data saturation, we used the following approach. We conducted data analysis after 16 interviews, rather than waiting until all interviews were completed. This allowed us to identify emerging themes early in the process. After the sixteenth interview, following every two to three interviews, we compared the codes and themes generated to those gathered from previous interviews. We determined that data saturation was achieved after the twenty-fourth interview, as the subsequent three interviews did not produce any new themes or significant insights. However, we conducted these final three interviews to ensure that all new themes existed in the previous data. Finally, a final data analysis was carried out.

### 2.2. Ethical Considerations

This study was approved by the College of Management Colman Ethics Committee and the methods and results reported in this article are presented according to the Consolidated Criteria for Reporting Qualitative Research (COREQ) guidelines [26]. Informed consent was obtained from all subjects involved in this study. All participants signed their consent form prior to the interview. Participants were informed of their right to withdraw from the study at any time without consequences. Participants were assigned unique identification codes, and all personal identifiers were removed from the transcripts. Audio recordings and transcripts were stored on password-protected, encrypted devices accessible only to the research team. Any potentially identifying information (e.g., specific workplace details) was generalized or removed during the analysis of results. It was emphasized that participation was entirely voluntary, and no incentives were offered. Participants were informed about how the results would be disseminated and were offered the opportunity to receive a summary of the findings.

### 2.3. The Interview Guides and Think-Aloud Technique

The interview guides were developed by a multidisciplinary team. First, based on our research questions and literature review, an initial draft was created by the primary investigators. Then, the draft was reviewed by our team, which included one senior self-employed FP and researcher, one junior self-employed FP, as well as two social and behavioral science and health policy researchers. Each member provided feedback based on their area of expertise. Throughout the data collection process, we held regular team meetings to discuss the effectiveness of the guide and made minor adjustments as needed to explore emerging themes.

The think-aloud technique was used to deepen and clarify the understanding of the interviewees’ pull and push factors. *Think-aloud* is a research method in which individuals verbalize their thoughts while performing a task and engage in decision making [27]. This technique was chosen because it does not increase the interviewee burden and may allow for the identification of more accurate push and pull factors [28]. Think-aloud data were collected in a concurrent manner, meaning responders were asked to verbalize their thoughts regarding their considerations to become self-employed after pilot think-a-loud prior research questions [24]. At the beginning of the interview, participants were briefed on the technique. We used prompts such as “Can you walk me through your thought process when you decided to become self-employed?” or “As you consider the pros and cons of self-employment, what goes through your mind?”. Participants were encouraged to verbalize their thoughts freely, without fear of judgment. The interviewer used minimal interruptions, only prompting when necessary to maintain the flow of verbalization. The full interview guides are provided in the Supplementary Materials. The interview guides covered several subjects beyond the scope of this article, addressing gender-related topics of self-employment.

### 2.4. Procedure

Thirty-two FPs agreed to participate, and a formal letter describing this study’s potential contribution to FP self-employed practice in Israel was sent to them. Finally, interviews were scheduled with twenty-seven FPs. Five doctors, although they agreed to be interviewed, ultimately could not be scheduled for interviews, likely due to their heavy workload. The interviews were conducted via Zoom meetings by the researchers and the research assistant, who is a professional group facilitator and recorded with the interviewee’s permission. Zoom was our main interviewing medium of choice because of its scheduling flexibility and geographic adaptability. Zoom interviews allowed us to include participants from diverse geographical locations without travel constraints and offered more flexibility in scheduling, accommodating the often-busy schedules of family physicians.

Interviews lasted between 40 and 110 min and were structured as follows. The introduction included a greeting, self-presentation, review of study purpose and consent, and explanation of the interview process and the think-aloud technique. The interview first focused on background information, professional history, and current practice. The topics included the decision to become self-employed, motivations for considering self-employment, the decision-making process (using the think-aloud technique), and reflections on career choice. Interviews ended with a closing question about additional thoughts.

The video Zoom file was deleted after the interview, and only the audio file was kept for transcription purposes and accuracy for later analysis as per Ethics Committee guidelines and terms of approval. This decision was based on data minimization principles and privacy protection. Full interview guides and the recruitment letter are available in the Supplementary Materials.

### 2.5. Data Analysis

Data analysis was performed inductively using a content analysis method [29] and divided into two themes: push and pull factors. We conducted the initial analysis midway through after the 16th interview, in order to apply the principle of data saturation and determine the final number of interviewees, as described in Section 2.1 above. First, the interview recordings were listened to, transcripts were read longitudinally and then laterally, and first impressions were documented separately by all researchers. Each researcher coded keywords and short phrases potentially representing themes and grouped them according to factor types and their influence (i.e., push or pull). Then, in a data analysis workshop, a comparative examination of the individual researchers’ analyses was performed, while looking for more connections and links among themes and discussing differences in identified themes. The researchers presented their individual analyses. They discussed each theme, explaining their own rationale and providing examples from the data. Themes with high agreement were retained and discrepancies were discussed in detail. For each discrepancy, researchers presented their perspectives. The team explored the context of each theme and its relevance to the research questions. Consensus was reached through open dialogue and mutual agreement. Similar themes were merged, and discrepancies were resolved through discussion. A board with movable notes was used by researchers to build a visual category tree. Researchers also shared their reflections and thoughts about self-employment and potential biases. Finally, a consensus was reached regarding the themes and their division into two main categories—push factors and pull factors. Two such workshops took place—one after the 16th interview and the second after the 24th interview. The final set of pull and push factors was reviewed in relation to the original research questions and the existing literature. Illustrative quotes were selected to support each theme.

## 3. Results

### 3.1. Family Physicians’ Characteristics

FPs working with four HMOs in Israel were fairly represented in this study. The FPs in the study sample also varied in age, gender, area of practice (center/periphery), type of practice (solo/group/HMO medical office buildings), and the patient population they serve (Hebrew, Arabic, and Russian speakers). The participants’ characteristics are displayed in Table 1.

Following interview data analysis, an overall number of two themes with ten sub-themes emerged. The FPs’ quotations are presented in Table 2. The two themes represent the push and pull factors that affected the FPs’ decision to become self-employed.

### 3.2. Theme One: Pull Factors

This theme includes those factors, described below, that pulled, motivated, and attracted FPs to become self-employed. The pull factors represent the anticipated improvements or advantages that FPs believe they will gain by transitioning to self-employment.

#### 3.2.1. Higher Income

Twenty-two out of twenty-seven of the interviewees mentioned the higher income potential of a private clinic compared to one managed by an HMO as a factor that attracted them to become self-employed. This financial incentive was multifaceted, with interviewees referring to both their absolute income and the “income relative to effort” and workload. The prospect of earning more for the same or similar work was appealing to many. Interestingly, the significance of higher income and financial benefits varied among the interviewees. For some, it was a primary motivator, representing a chance to significantly improve their financial situation. For others, while still important, it was one of several factors influencing their decision to transition to self-employment. This variation highlights the complex interplay of financial and non-financial factors in FPs’ career decisions.

#### 3.2.2. Professional Self-Fulfillment and Identity

This theme describes how self-employment aligns with and facilitates the core values and professional identity of family physicians (FPs). Out of 27 interviewees, 20 expressed that self-employment settings provided a higher ability to realize professional “family medicine values”. This concept encompasses the fundamental principles that guide family medicine practice, such as continuity of care, patient-centered approach, and holistic health management. These values were raised as being linked to FPs’ professional identity and sense of purpose. Interviewees shared personal beliefs and assumptions that shaped their identity as family physicians, reflecting on how self-employment allowed them to practice medicine in a way that aligns more closely with their ideals. This alignment between practice environment and professional values was seen as crucial for job satisfaction and a sense of authenticity in their work. The freedom to implement these values without institutional constraints was frequently cited as a significant advantage of self-employment.

#### 3.2.3. Professional, Organizational, and Business Autonomy

Twenty-six out of twenty-seven respondents cited autonomy as a pull factor for becoming self-employed. They referred to various aspects of this autonomy, including professional, organizational, and business autonomy.

Professional autonomy, from the FPs’ perspective, referred to the “possibility of making ‘pure’ medical decisions” in relation to the required diagnostic procedures and treatment methods, as well as the freedom to base patients’ treatment decisions solely on “medical-only” concepts and understandings.

Organizational autonomy referred to the FPs’ choices regarding how they organize work in their clinic “according to their own ideas and understandings”. This included appointment schedules, telemedicine work processes—mainly in store-and-forward messages and phone calls, and dealing with a variety of patient populations, such as chronic patients, young patients, and more.

Business autonomy included references to the ability to have a high impact on profit and a combination of different ways of earning, including decisions about the number of working hours, the execution of various operations, renting doctors’ rooms, managing expenses and income, and related occupations that were perceived as more possible in independent medicine, such as combining their medical practice work with various start-ups, investments, etc.

#### 3.2.4. Having My Own Secretary

In sixteen interviews, the role of the secretary was mentioned as fundamental in the clinic, as the secretary can “influence all aspects of the clinic”, including income, the order and organization of processes, “climate in waiting room”, and the doctor’s “quality of life”. Interviewees emphasized that a competent secretary can significantly impact the clinic’s financial performance through efficient scheduling and billing practices, enhance operational efficiency by managing patient flow, and contribute to a positive environment with patients through their interpersonal skills. Therefore, being able to choose one’s own secretary was mentioned in the interviews as a pull factor, unlike in HMO clinics, where “secretaries are loyal to the HMO, rather than the FP”. This autonomy in selecting administrative support aligned with the physician’s values, and work style was seen as a crucial advantage of self-employment, potentially leading to better alignment of goals and improved overall practice management.

#### 3.2.5. Designing the Physical Space of the Clinic

The ability to design the physical work environment according to the FP’s preferences was mentioned in 19 interviews as a factor that attracted the FPs to open a private clinic. Some interviewees emphasized the connection they see between the physical space of the clinic and the patients’ and FPs’ experience of the treatment provided, highlighting how the environment can influence the quality of care and patient–FP interactions. Others expressed satisfaction in independently designing the clinic space according to their own personal preferences, without having to negotiate over issues related to the space with the HMO. Some interviewees specifically referred to the interior design of the clinic and to the building itself—whether the clinic is in a quiet, small space or in a busy HMO building with many doctors’ offices. This choice of location and setting was seen as crucial in shaping the practice atmosphere and patient experience. FPs appreciated the ability to choose between a more intimate, personalized setting and a larger, more bustling environment based on their practice philosophy and patient needs.

#### 3.2.6. Flexible Working Hours

In 15 out of 27 interviews, the topic of flexible working hours, which allows for a better work–home balance, emerged as a pull factor towards self-employment. This aspect of self-employment was seen as a significant advantage over the often-rigid schedules associated with HMO employment. Interviewees emphasized how this flexibility enabled them to better manage their personal and professional lives, leading to improved overall well-being and job satisfaction. Some FPs noted that the ability to set their own hours allowed them to accommodate personal commitments, such as family responsibilities, without compromising their professional obligations. This flexibility was particularly valued by FPs having young children. Others mentioned how it allowed them to align their work hours with their natural productivity rhythms, potentially leading to more efficient and effective patient care.

### 3.3. Theme Two: Push Factors

This theme encompasses the various elements that demotivated family physicians (FPs) from choosing or continuing HMO employment, consequently pushing them towards self-employment. These factors represent the challenges, frustrations, and limitations FPs experienced or perceived within the HMO setting that made self-employment appear as a more attractive alternative.

#### 3.3.1. Low Control over the Work Environment

Twenty-two out of twenty-seven interviewees mentioned the low control over the work environment in HMOs as a significant push factor towards self-employment. This low level of control manifested in various aspects of their daily practice, creating a sense of frustration and professional constraint. They referred to the limited ability of HMO-employed FPs to design their work diaries, determine the duration of patient visits, or manage the mixture of patient types according to their own preferences (such as balancing chronic conditions, pediatric care, or geriatric patients).

#### 3.3.2. Tensions and Conflicts with the HMO’s Non-Medical Staff

Eighteen out of twenty-seven participants mentioned the tensions and conflicts between non-medical management teams and how the management teams served as a cause of dissatisfaction and a push factor not to stay or not to choose to be an HMO-employed FP. These tensions often arose from differing priorities and perspectives between medical professionals and administrative staff, leading to frustration and a sense of professional compromise. Interviewees described situations where administrative decisions conflicted with what they perceived as being the best medical practices, creating ethical dilemmas and stress. They also mentioned conflicting goals between business and medical purposes of tasks as in cases when the management expects FPs to promote an increase in the patient population, be engaged in HMO marketing, and other business management tasks versus pure medical goals. This divergence in objectives was seen as particularly problematic, with FPs feeling pressured to prioritize organizational tasks over patient care tasks. Some interviewees expressed discomfort with being asked to participate in HMO activities they viewed as being more aligned with business development than healthcare provision, feeling that such expectations detracted from their core mission as medical professionals.

#### 3.3.3. Decreased Professional and Organizational Autonomy

Fourteen FPs interviewed suggested that managerial processes and supervision in HMOs sometimes harmed their autonomy and mentioned this as a push factor to stop working with HMOs and consider self-employment. This perceived erosion of professional autonomy was raised as a source of frustration for FPs, who felt that standardized protocols often interfered with their ability to make individualized clinical decisions. Interviewees described feeling constrained by rigid organizational policies that did not always align with their professional judgment or the unique needs of their patients. Ten FPs mentioned particularly the control processes and feedback related to medical quality measures. While acknowledging the importance of quality assurance, many FPs felt that the implementation of these measures was often overly prescriptive and failed to account for the nuances of patient care. They expressed frustration with performing evaluations that prioritized quantitative metrics over qualitative aspects of care, feeling that such approaches oversimplified the complex nature of family medicine. This emphasis on standardized measures was seen by some as undermining their clinical expertise and professional discretion, ultimately pushing them to consider self-employment as a means to regain control over their practice and decision-making processes.

#### 3.3.4. Workload and Working Conditions

Most of the participants, specifically 23 out of 27, pointed out the issues of workload and working conditions, including shortages in personnel, as a significant push factor toward self-employment. The overwhelming patient load, often coupled with insufficient time allocated per patient, was frequently cited as a major source of stress and job dissatisfaction. Interviewees described a feeling of constantly being rushed, unable to provide the level of care they believed their patients deserved, and experiencing burnout as a result of these demanding conditions. Ten FPs specifically mentioned inadequate secretarial services as a push factor towards self-employment. The frustration stemmed from the fact that secretaries are also HMO-employed, leading to a perceived lack of loyalty or dedication to individual FPs. As one participant colorfully described, they are not “really (FPs’) personal secretary, they are like a clerk that guides people to the right room”. This sentiment reflects an issue raised in these interviews of misalignment between administrative support and the needs of medical professionals. The findings described above reveal a complex interplay of pull and push factors. Pull factors attracting FPs to self-employment include increased professional autonomy, higher income potential, control over work environment, and flexible working hours. Push factors driving FPs away from HMO employment include low control over the work environment, excessive workload and poor conditions, managerial interference with autonomy, and tensions with non-medical staff. These results suggest that while financial incentives are important, issues of professional autonomy, work environment control, and dissatisfaction with HMO management practices are equally, if not more, significant in driving the trend towards FPs’ self-employment.

## 4. Discussion

This study explored the push and pull factors that influence FPs in Israel to choose self-employment, contributing novel insights to the existing literature on physician employment choices. Our research identifies the process of increasing bureaucratization and loss of professional autonomy as a significant push factor towards self-employment, a finding not previously highlighted in this context. We provide new evidence on the impact of tensions between FPs and non-medical management in Health Maintenance Organizations (HMOs) as a push factor toward self-employment. Our study also offers a perspective on the evolving priorities of younger FPs, particularly regarding work–life balance and professional autonomy. These findings extend beyond the commonly discussed factors of income and general autonomy, providing a more nuanced perspective of the decision-making process FPs go through when considering self-employment.

Higher income, self-fulfillment, increased autonomy, the ability to choose one’s own secretary, designing the clinic space, and enjoying flexible working hours were all identified as pull factors towards FPs choosing self-employment. Push factors included low control over the work environment, workload in HMOs, inadequate secretarial services, decreased professional and organizational autonomy, and tensions with the HMO’s non-medical staff.

In line with the existing literature, higher income and increased professional autonomy are common factors for FPs’ self-employment [19,20]. In this study, as in several other recent studies [30,31], regarding the income factor, the “reasonable income-to-lifestyle ratio” was especially emphasized. This reflects the preferences of the younger generation of FPs to have a controllable lifestyle that allows them to spend time with their family while managing their medical practice [32].

Push factors referred to decreased autonomy and significant managerial control over the work environment, tight supervision in HMOs, tensions between non-medical managers and managers’ expectations and requests from doctors to engage in marketing. The fact that these issues are a significant part of FPs’ push factors for self-employment is a new and important finding. It is consistent with recent studies from Israeli and other primary health systems that indicate a widening power gap between doctors and non-medical managers [33,34,35].

Taking a holistic view of these push factors, it seems that the majority of them present different facets of the same phenomenon—the *proletarianization* of the medical profession that takes place, especially among salaried, HMO-employed FPs. Proletarianization of medicine refers to a process occurring in medical organizations “as a result of the bureaucratization which is being forced on medical practices as a consequence of the logic of capitalist expansion” [36,37]. As a result of this process, “physicians are slowly being reduced to a proletarian function, and their formerly self-interested activities are subordinated to the broader requirements of the capitalist control of highly profitable medical production” in current health organizations [37,38].

The proletarianization of the medical profession, as is evident among family physicians employed by HMOs, presents both functional and ethical challenges. Functionally, FPs often view bureaucratic tasks as time-consuming and detrimental to patient care. For instance, the requirement to engage in marketing activities or meet externally set quality indicators often conflicts with FPs’ primary focus on patient welfare. In the ethical realm, employed FPs frequently face a double-agent dilemma, torn between their commitment to patient-centered care and the operational efficiencies demanded by HMOs.

Medical professionalism, which encompasses a commitment to the welfare of patients, adherence to ethical principles, fostering of a patient-centered approach, and being an advocate of the patient [39,40], often clashes with HMOs’ focus on operational efficiencies and cost containment. This misalignment creates a significant push factor towards self-employment, as FPs seek to resolve the ethical dissonance by freeing themselves from corporate obligations that may conflict with their professional values.

Conversely, HMOs, as organizational entities, are often oriented towards operational efficiencies, cost containment, and meeting externally set quality indicators, which may not always align with the tenets of medical professionalism and the needs of the individual patient. This predicament thrusts FPs into the demanding role of serving “two masters”. Choosing a self-employed practice can provide a remedy to this ethical dissonance by freeing FPs from the obligation to prioritize HMO corporate interests. Additionally, the FPs’ engagement in administrative responsibilities, such as reporting, could contribute to professional dissatisfaction and burnout among FPs, as it detracts from time spent on direct patient care and may not align with their intrinsic motivations for practicing medicine [41].

This finding is new and significant in understanding why FPs in different markets choose self-employment. To the best of our knowledge, this finding was not previously described in the literature as a push factor, despite its important implications for policy makers.

Our findings strongly support and extend the theory of proletarianization in the medical profession, particularly as it applies to family physicians in managed care settings. The push factors identified in our study align closely with the core concepts of proletarianization theory. Our study extends this theory by providing concrete examples of how proletarianization manifests in the daily experiences of FPs, such as the pressure to engage in marketing activities and meet externally set quality indicators that may not align with their professional judgment. Furthermore, our findings suggest that proletarianization is not just a passive process that physicians undergo, but rather an active driver of career decisions, pushing FPs towards self-employment as a means of reclaiming their professional autonomy. This adds a new dimension to the theory, highlighting how proletarianization can influence workforce trends and healthcare delivery models.

Our findings have important implications for healthcare policymakers and HMO managers. To retain FPs within the HMO employment framework, organizations must address the issues of non-medical management pressures and reduced autonomy. We propose designing HMOs as more loosely coupled systems [42,43], characterized by reduced medico–bureaucratic regulations, limited non-medical supervision, the decentralization of power, and greater alignment of organizational structures with clinical tasks. Specific changes could include implementing flexible scheduling systems that allow FPs more control over their work hours, reducing administrative burdens by streamlining paperwork processes, and involving FPs in decision-making about clinical protocols and quality metrics. HMOs could also establish physician-led committees to mediate between clinical and administrative priorities, ensuring that FPs’ voices are heard in organizational decisions. These changes could create more balanced work environment structures, which will be able to retain FPs within the HMO employment framework.

### Limitations

This study has its limitations. First, as a qualitative study, our findings are based on in-depth experiences of a relatively small sample of family physicians in Israel, which may limit the generalizability of the results to broader populations or different healthcare contexts.

Additionally, while our approach allowed for rich, detailed insights into the push and pull factors influencing FPs’ career choices, it does not provide quantitative measurements of the relative importance or prevalence of these factors across the entire FP population.

Another limitation is related to HMO representation in the sample. There is a strong representation of self-employed FPs working with Maccabi HMO (70%) in our sample, which reflects the actual distribution of self-employed FPs across Health Maintenance Organizations in Israel. Indeed, among all HMOs, Maccabi (Ma) has the highest number of agreements with self-employed physicians. However, this significant representation of physicians working with Maccabi in our sample may introduce a potential bias. While it accurately represents the current landscape, it might lead to an overemphasis on experiences and perspectives specific to Maccabi’s policies, practices, and work environment. This could potentially underrepresent the unique challenges experienced by self-employed FPs working predominantly with other HMOs in Israel.

While our study primarily reflects the Israeli healthcare system, similar trends have been observed in other countries. Studies in the United States, United Kingdom, and Canada have reported increasing dissatisfaction among employed physicians due to loss of autonomy and bureaucratic burdens [43,44,45]. These parallels suggest that the push and pull factors identified in our study may have broader applicability. However, the extent and specific manifestations of these trends likely vary depending on the unique characteristics of each healthcare system, such as the degree of privatization, strength of primary care infrastructure, and cultural attitudes towards medical professionalism.

Future comparative research could provide valuable insights into global health policy, potentially identifying the best practices for balancing organizational efficiency with physician satisfaction and autonomy across different healthcare models. Future research should also focus on developing and testing specific interventions to address the push factors identified in this study. Key areas for investigation include evaluating FP-led committees in HMOs that mediate between clinical and administrative priorities, assessing tiered autonomy systems for experienced FPs, investigating flexible scheduling systems, studying the effects of reduced administrative burdens, and exploring mentorship programs for younger FPs. Additionally, longitudinal studies tracking FPs transitioning to self-employment and cross-cultural comparative studies could provide valuable insights into long-term career satisfaction and the influence of different healthcare systems on FPs’ employment preferences.

## 5. Conclusions

Our study reveals that family physicians (FPs) in Israel are increasingly drawn to self-employment due to a complex interplay of push and pull factors. FPs seek environments offering greater professional autonomy, flexible working hours, and practice control, while administrative pressures, excessive bureaucracy, and non-clinical tasks drive them away from HMO employment. These findings highlight the tension between medical professionalism and organizational demands in HMOs. To retain FPs, healthcare policymakers and HMO managers must address these issues by redesigning organizations as more loosely coupled systems with reduced medico–bureaucratic regulations and greater alignment with clinical priorities. Understanding these factors is crucial for effective workforce planning, resource allocation, and creating balanced work environments that respect FPs’ professional judgment while meeting organizational goals. This approach is essential not only for retaining FPs within HMOs but also for ensuring effective collaboration with self-employed FPs, ultimately contributing to more robust and responsive healthcare systems.

## Figures and Tables

**Table 1:** Family physicians’ characteristics (N = 27).

Characteristics	N (%)
Gender, Female	18 (67%)
Mean Age, years	42.6
Marital Status, married	24 (88%)
Number of children	2.4
Specialization, Family Medicine **	24 (87%)
Years since Family Medicine specialization graduation, mean	6
Geographic area of practice	North 4 (15%), Center 16 (59%), Jerusalem 5 (19%), South 2 (7%)
Family Medicine specialization organization ***	Ma 14 (55%), Me 6 (22%) C 4 (14%), L 1 (1%), other 2 (1%)
Language	Hebrew (100%), Arabic (30%), Russian (20%)
Work with Health Maintenance Organization *	Ma 19 (70%), Me 5 (19%) C 2 (7%), L 0 (0%), MTO 5 (18%)

* C = Clalit, Ma = Maccabi, Me = Meuhedet, L = Leumit, MTO = More than one health maintenance organization. ** General practitioners work as family physicians in certain HMOs in Israel (especially Meuhedet and Leumit). *** Family Medicine specialization organization: the HMO where FP completed their Family Medicine residency training. Work with Health Maintenance Organization: the HMO(s) FP currently has a working agreement as a self-employed physician.

**Table 2:** Overview of themes and sub-themes.

Themes	Definition	Sub-Themes	FPs’ Quotations
**Pull Factors**	This theme includes factors that pulled, motivated, and attracted family physicians to become self-employed.	Higher income	“In private [family] medicine, the income can be several times higher than in an HMO *, and this strengthened my decision to be self-employed”. *“The first and central thing about being self-employed is the salary options”.* *“Everyone is busy with primary care; so if I work hard, at least I earn more for it and I know why I do it”.*
		Professional self-fulfillment	*“It’s just who I am, I’m not a system person, I don’t see myself at all working as a doctor in one HMO or another”.* *“It was clear to me from the beginning that I would be a self-employed physician. By nature, I am kind of a ‘soloist’. I hate systems, I hate bosses, I hate HMOs’ logos […] As far as I’m concerned, I work for patients, not for one organization or another”.* *“The economic benefits are definitely there (in private practice), but I’m not even sure that was the main consideration [to becoming self-employed]. To be your own boss and not to be subject to the caprices of the system—it’s a part of me, it’s always been important to me”.*
		Professional, organizational, and business autonomy	*“You practice medicine the way you think it should be done, you determine how long your queue is, you determine which days you will work, you determine when you will take time off, you really determine your priorities in the clinic and [in the way you practice] medicine. As an employee, you work in the branch and are subject to all kinds of system nonsense”.* *“As a self-employed person, I’m really ‘my own master’ [regarding practicing] medicine and my appointments. If I decide that my appointment is fifteen minutes long, which is a crazy luxury in family medicine, and I only register four people per hour to begin with, then that’s how it’s going to work. I decide, and no one can tell me anything”.* *“You run your own business and pay your own rent and the secretary’s salary, and all the maintenance and cleaning. You have a lot of expenses, but they are your own decisions, and you make them”.* *“For the self-employed [FPs], the thinking is very different from the salaried ones. I can think about how to expand the clinic, maybe rent out rooms. I feel that in the future I’ll be able to take care of the income—not only as a doctor who receives patients, but also to develop other sources of income. That’s impossible when you’re an HMO employee”.*
		“Having my own secretary”	*“A lot depends on the secretary”.* *“If I chose her, and I trained (?) her, then her commitment is to me and to me only. The adaptation to my needs will be also higher. …. and she will be loyal to me …rather than to the organization”.*
		Designing the clinic’s physical space	*“In terms of design, it was important to me to have a place that is not identified with any HMO, but is identified with humanity in general. I wanted my own colors and not HMO branding colors”.* *“The whole thing about the clinic’s design was important to me. I have the option to decide if I think there should be a window in the room”.* *“I told the HMO that if I start my own clinic, I want it to be my clinic with my photos and my furniture, and I want to feel at home here. It was important to me that the place would not be alienating, and that people would feel comfortable”.*
		Flexible working hours	*“When I decide to take a vacation, I close my diary and take time off, and it’s just my decision”.* *“My wife is a surgeon, so I am a full-time father. I need the flexible working hours that an independent practice can provide”.*
**Push Factors**	This theme includes factors that demotivated family physicians to choose HMO employment and pushed them to become self-employed.	Low control over the work environment	*“In our (HMO) district, every secretary could close my diary, reserve hours. I myself couldn’t close off hours in my diary for answering the digital messages from patients, for example”.* *“When I was an [X] HMO employee, if I wanted to allocate my time differently, or to devote more time to certain patients, [I couldn’t]. The system dictated what I could do. Sometimes, I felt like a small cog in a big system, with very little personal control”.* *“This is also something I couldn’t do as an employee—control the waiting times, the duration of the visits, for example, if I wanted to allow three or four urgent visits at the end of the day for really small things”.*
		Tensions with the HMO’s non-medical staff	*“Even during the busiest periods, physicians are expected to engage in capitation and growth, [and help recruit more patients to the HMO]. I didn’t go to study medicine to engage in marketing, it doesn’t interest me”.* *“Competition between the HMOs (patients’ growth) is none of my business”.* *“Those who ‘set the tone’ in these HMOs are non-medical managers. It comes from the top down and you feel it in everything. It was unbearable”.* *“What mainly created the chaos [when I was working in the HMO] and the impossible working conditions isn’t really the workload—which is something objective—but the fact that the non-medical managers didn’t make any effort to make it easier for us. On the contrary, they would disturb us”.*
		Decreased professional and organizational autonomy	*“My professional autonomy was low. It was a combination of my control over the diary, my control over the medicine and the quality of the medicine—all of those were at a low level. My influence on the team was also low”.* *“There were times when the pressure from managers on quality indicators was unbearable. They are not interested in hearing what you have to say, and just constantly exert pressure. They say that now it has calmed down [in HMOs], but this whole way of working didn’t suit me”.*
		Workload and working conditions	*“I had unreasonable workloads when I was an [HMO] employee. [In the HMO], it very much depends on the clinics and the personnel who sit there, but basically there is a lack of personnel, there are very few secretaries for many FPs, they have less time, and the quality of the personnel is also very variable”.* *“The working conditions in general were poor. They were difficult, the workload was inhumane, and no one made any effort to help us provide the patients with good medical services. At some point, it became so extreme in one of the clinics where I worked, that I raised it for discussion with the management. Instead of listening to what I had to say, and trying to help, they made it even more difficult for me. It made me want to ‘keep my head down’ even more”.*

* HMO—Health Maintenance Organization.

## Data Availability

The original contributions presented in this study are included in the article; further inquiries can be directed to the corresponding authors.

## Supplementary Materials

The following supporting information can be downloaded at: https://www.mdpi.com/article/10.3390/healthcare12171749/s1, Full interview guides and recruitment letter.

## References

[1] Kovach K.A., Reid K., Grandmont J., Jones D., Wood J., Schoof B. (2019). How engaged are family physicians in addressing the social determinants of health? A survey supporting the American academy of family physician’s health equity environmental scan. Health Equity.

[2] Brekke K.R., Sørgard L. (2007). Public versus private health care in a national health service. Health Econ..

[3] Pearlman A. (2000). The Rise of Managed Care and the Decline of Physician Self-Employment. https://www.semanticscholar.org/paper/The-Rise-of-Managed-Care-and-the-Decline-of-Pearlman/9a75230d19a3887ed57585e7003b577142169f5f.

[4] Zyzanski S.J., Gonzalez M.M., O’neal J.P., Etz R.S., Reves S.R., Stange K.C. (2021). Measuring primary care across 35 OECD countries. Ann. Fam. Med..

[5] Iupati S., Stanley J., Egan R., MacLeod R., Davies C., Spence H., Iupati D., Middlemiss T., Gwynne-Robson I. (2023). Systematic review of models of effective community specialist palliative care services for evidence of improved patient-related outcomes, equity, integration, and health service utilization. J. Palliat. Med..

[6] U.S. Department of Health and Human Services (2016). State-Level Projections of Supply and Demand for Primary Care Practitioners: 2013–2025.

[7] Khalil-Khan A., Khan M.A. (2023). The Impact of COVID-19 on Primary Care: A Scoping Review. Cureus.

[8] Prunuske J. (2020). America needs more family doctors: The 25 × 2030 collaborative aims to get more medical students into family medicine. Am. Fam. Physician.

[9] Ashmore J. (2013). ‘Going private’: A qualitative comparison of medical specialists’ job satisfaction in the public and private sectors of South Africa. Hum. Resour. Health..

[10] Ashton T., Brown P., Sopina E., Cameron L., Tenbensel T., Windsor J. (2013). Sources of satisfaction and dissatisfaction among specialists within the public and private health sectors. N. Z. Med. J..

[11] Association of American Medical Colleges The Complexities of Physician Supply and Demand: Projections from 2016 to 2030. https://aamc-black.global.ssl.fastly.net/production/media/filer_public/85/d7/85d7b689-f417-4ef0-97fb-ecc129836829/aamc_2018_workforce_projections_update_april_11_2018.pdf.

[12] Ben Naim G., Hershko D. (2022). The Salary of Family Doctors in the Health Funds—Self-Employed versus Employees, 2010–2018. Israel Ministry of Finance. https://www.gov.il/BlobFolder/reports/article_07092022/he/Publishes_Articles_article_07092022.pdf.

[13] Chernichovsky D. (2019). The Healthcare System: An Overview.

[14] Tur-Sinai A., Zontag N., Blondheim O., Weinreb A., Chernichovsky D. (2020). Physicians in Israel: Trends in characteristics and training. State of the Nation Report: Society, Economy and Policy in Israel.

[15] Waitzberg R., Quentin W., Webb E., Glied S. (2021). The Structure and Financing of Health Care Systems Affected How Providers Coped With COVID-19. Milbank Q..

[16] Tabenkin H., Gross R. (2000). The role of the primary care physician in the Israeli health care system as a ‘gatekeeper’—The viewpoint of health care policy makers. Health Policy.

[17] Lee E.S. (1966). A theory of migration. Demography.

[18] Kirkwood J. (2009). Motivational factors in a push-pull theory of entrepreneurship. Gend. Manag. Int. J..

[19] Ogundeji Y.K., Quinn A., Lunney M., Chong C., Chew D., Danso G., Duggan S., Edwards A., Hopkin G., Senior P. (2021). Factors that influence specialist physician preferences for fee-for-service and salary-based payment models: A qualitative study. Health Policy.

[20] Behmann M., Schmiemann G., Lingner H., Kühne F., Hummers-Pradier E., Schneider N. (2012). Job satisfaction among primary care physicians: Results of a survey. Dtsch Arztebl. Int..

[21] American Academy of Family Physicians (2021). 2021 AAFP Member Profile Survey. https://www.aafp.org/news/health-of-the-public/2022-nrn-annual-report.html.

[22] Sinsky C., Linzer M. (2020). Practice and Policy Reset Post-COVID-19: Reversion, Transition, Or Transformation?. Health Aff..

[23] The Physicians Foundation (2022). 2022 Survey of America’s Physicians: COVID-19 Impact Edition. https://physiciansfoundation.org/research/the-physicians-foundation-2021-physician-survey/.

[24] Croswell J.W. Research Design: Quantitative, Qualitative Research and Mixed Methods Approaches. Third Edition. https://www.ucg.ac.me/skladiste/blog_609332/objava_105202/fajlovi/Creswell.pdf.

[25] Tong A., Sainsbury P., Craig J. (2007). Consolidated criteria for reporting qualitative research (COREQ): A 32-item checklist for interviews and focus groups. Int. J. Qual. Health Care.

[26] Ericsson K.A., Simon H.A. (1984). Protocol Analysis: Verbal Reports as Data.

[27] Peterson C.H., Peterson N.A., Powell K.G. (2017). Cognitive interviewing for item development: Validity evidence based on content and response processes. Meas. Eval. Couns. Dev..

[28] Willis G.B. (2004). Cognitive Interviewing: A Tool for Improving Questionnaire Design.

[29] Goldman A.L., Barnett M.L. (2023). Changes in physician work hours and implications for workforce capacity and work-life balance, 2001–2021. JAMA Intern. Med..

[30] Kohnstamm R.R. (2022). Integrative Wellness Coaching: Physician Self-Care and Personal Insight Are Critical for Improved Work/Life Balance. Integr. Complement. Ther..

[31] Naimer S., Press Y., Weissman C., Zisk-Rony R.Y., Weiss Y.G., Tandeter H. (2018). Medical students’ perceptions of a career in family medicine. Isr. J. Health Policy Res..

[32] Chudner I., Drach-Zahavy A., Goldblatt H., Goldfracht M., Karkabi K. (2020). Power Gaps Among Stakeholders in Israel’s Primary Care and the Role of Primary Care Physicians’ Relative Power in Their Intention to Use Video-Consultations with Patients. Telemed. e-Health.

[33] Silver J.K., Bean A.C., Slocum C., Poorman J.A., Tenforde A., Blauwet C.A., Kirch R.A., Parekh R., Amonoo H.L., Zafonte R. (2019). Physician workforce disparities and patient care: A narrative review. Health Equity.

[34] Gask L. (2004). Powerlessness, control, and complexity: The experience of family physicians in a group model HMO. Ann. Fam. Med..

[35] McKinlay J.B., Arches J. (1985). Towards the proletarianization of physicians. Int. J. Health Serv..

[36] Coburn D. (2021). Canadian medicine: Dominance or proletarianization?. The Corporate Transformation of Health Care.

[37] Light D., Levine S. (2019). The changing character of the medical profession: A theoretical overview. Management of Healthcare.

[38] Cruess S.R., Cruess R.L., Steinert Y. (2019). Supporting the development of a professional identity: General principles. Med. Teach..

[39] Laine C., Davidoff F. (1996). Patient-centered medicine: A professional evolution. JAMA.

[40] Shanafelt T.D., Dyrbye L.N., West C.P., Sinsky C.A. (2016). Potential impact of burnout on the US physician workforce. Mayo Clin. Proc..

[41] Burke W.W. (2014). Changing loosely coupled systems. J. Appl. Behav. Sci..

[42] Sinsky C.A., Biddison L.D., Mallick A., Dopp A.L., Perlo J., Lynn L., Smith C.D. (2020). Organizational Evidence-Based and Promising Practices for Improving Clinician Well-Being. NAM Perspect..

[43] Shanafelt T.D., Noseworthy J.H. (2017). Executive Leadership and Physician Well-being: Nine Organizational Strategies to Promote Engagement and Reduce Burnout. Mayo Clin. Proc..

[44] Dale J., Potter R., Owen K., Parsons N., Realpe A., Leach J. (2015). Retaining the general practitioner workforce in England: What matters to GPs? A cross-sectional study. BMC Fam. Pract..

[45] Malko A.V., Huckfeldt V. (2017). Physician employment in health systems: A growing trend. J. Med. Pract. Manag..

