# Supplementary material for: Becoming Self Employed: Israeli Family Physicians’ Push and Pull Factors
Source: Healthcare (Basel). 2024 Sep 2;12(17):1749. doi: 10.3390/healthcare12171749 (PMC11394856; doi:10.3390/healthcare12171749)
Supplement: Supplementary file 1 [file healthcare-3135426-supplementary.pdf › healthcare-3135426-supplementary.pdf]

הפקולטה לרפואה ע"ש סאקלר
אוניברסיטת תל אביב

Jan-Feb 2022

Request to participate a personal interview as part of the study

"Self-Employed family medicine clinic"

Dear Dr. ____________,

The study "Self-employed family medicine clinic" examines Israeli family doctors' choice to operate under a self-employed practice model, and we would want you to participate in it.

In this phase of the study, we are interviewing family doctors who are self-employed. Your experiences, knowledge, and insights will be invaluable in helping us to comprehend the subject, which is why we are reaching out to you to set up a time for a face-to-face Zoom interview. We sincerely hope that you will agree to this and ready to cooperate.

The research representative will contact you shortly

Thanks in advance,

The research team

Interview guide

The Interview guides were created in Hebrew and translated to English for the purposes of this article

Primary Care Physicians Individual Interview Guide

Opening for individual interview

Thank you for agreeing to participate in this interview, which we are conducting as part of a study on primary care physicians' practice models in Israel. The purpose of the study is to examine the factors that influence the choice of being self-employed physician. I would like to interview you about your perceptions, as a self-employed primary care physician and your participation in the study may contribute significantly to understanding this issue. The interview will last an hour and a half.

Anything you share in the interview will be confidential and will be used only for research purposes. With your consent, I will record the interview, which will be circulated later.

[If agreed, recording is starting now]

From this point forward, and throughout the study, confidentiality will be preserved by not disclosing any information that can identify you and individually link you to what was mentioned during the interview.

Your name will not be included in the recording and transcript of the interview. The data will be analyzed according to different subjects and not by interviewees, to protect the confidentiality. Therefore, it will not be possible to identify you from the findings of the study.

I'll ask you a number of questions during the interview in an effort to get information from you regarding the subject. There are no right or wrong answers in this case; what matters is how you interpret the situation.

I will respect you even if I ask you questions you would rather not answer or discuss subjects you would rather not talk about. You have the option to withdraw from an interview at any time. I'll honor your request and won't take any adverse action against you.

Would you like to ask me something about the framework of this interview? *[If not, start the interview]*

Questions Interview Guide

- Tell me about yourself, your clinic and your decision to work in a self-employed model (solo, group practice/self-employed inside the HMO facilities).
- When and where did you graduate the Family Physician Specialty program?

[*Think a-loud technique opening*]

Using a special technique known as the "think-a-loud technique," I would like you to share with me how you came to the decision to work as a self-employed physician or as an employee of an HMO. Essentially, I'm asking you to speak aloud what was going through your mind when you made the decision, as well as the pros and cons of each option you looked at.

In order to check whether it is appropriate and if you can also respond to interview questions for me using this way, may I also advise that you practice this unique method by asking you a different question unrelated to the topic of the interview?

"Make an effort to picture your home and consider the number of windows it contains. Tell me what you are seeing and considering while you count the windows."

[Depending on how well the interviewee complete this exercise, further guidance may be necessary, prior to beginning the core part of the interview. If done go to prompt Think a loud question]

• Now that we've practiced "think aloud question and answer," could you kindly tell me what factors you took into account when choosing between working as a self-employed physician or as a hired physician by an HMO? What were the benefits and drawbacks of each choice?

[Choose a factor/consideration mentioned by an interviewee and deepen the understanding of this or that factor]

- What if a new physician, just starting his family medicine specialty program, approached you for advice on which practice model would be best for him to use right now? What would you advise regarding him?
- Is there anything more you feel is necessary to add to the discussion of these subjects that hasn't been mentioned?
- Thank you very much for your participation in the interview!

המכללה למינהל
המסלול האקדמי

הפקולטה לרפואה ע"ש סאקלר
אוניברסיטת תל אביב

Personal details

Number of interviewee _______________ Date _______________

Personal Information Gender: (1) man, (2) woman (3) other (4) prefer not to tell

Age ____________ Country of medical school graduated (1) Israel (2) Eastern Europe (3) Western Europe

Geographical Region of your clinic (1) North (2) Haifa (3) Hasharon (4) Center (5) Jerusalem (6) South

Place of HMO you graduated Family Medicine specialty program___________

HMO you work with as s self-employed (1) Clalit, (2) Maccabi, (3) Meuhedet, (4) Leumit (5) other

Patients’ population in my clinic speaking (1) Hebrew (2) Arabic (3) Russian (4) other ___ (mark all relevant answers)
